# Application of optimized digital surgical guides in mandibular resection and reconstruction with vascularized fibula flaps: Two case reports

**DOI:** 10.1097/MD.0000000000021942

**Published:** 2020-08-28

**Authors:** Lu Han, Xiaojie Zhang, Zeyou Guo, Jie Long

**Affiliations:** a The State Key Laboratory of Oral Diseases; b Department of Oral and Maxillofacial Surgery, West China College of Stomatology, Sichuan University; c National Engineering Laboratory for Oral Regenerative Medicine; d Stomatology Hospital, Zhejiang University School of Medicine; e Engineering Research Center of Oral Translational Medicine, Ministry of Education, Chengdu, P.R. China.

**Keywords:** digital surgical guides, mandibular reconstruction, preformed technology, preoperative plan

## Abstract

**Rationale:**

Currently, digital surgical techniques have been widely used in the precise treatment of mandibular resection and reconstruction with fibula flaps. Utilizing these innovative techniques in surgical planning and hardware fabrication before surgery has shown to provide great help. However, it is difficult for even experienced surgeons to place the preformed reconstruction plate in the same position as its preoperative design, causing surgical results to differ from preoperative planning. This study aims to solve these acknowledged challenges by creating newly designed equipment.

**Patient concerns:**

Two patients suffering from long-term expansion of the mandible were admitted to our department. Case I was a 39-year-old female patient who was concerned about the disease in the middle of the mandible, Case II was a 45-year-old female patient who was concerned about the disease at the left mandibular angle and ramus region.

**Diagnoses:**

Two patients were diagnosed with the mandibular ameloblastoma based on computed tomography (CT) scan and pathological results.

**Interventions:**

Personalized 3-dimensional (3D) surgical guides were applied to 2 patients with mandibular ameloblastoma who underwent mandibular resection and reconstruction with vascularized fibula flaps using a specially optimized and designed reconstruction guide plate.

**Outcomes:**

We achieved precise mandibular repair with such a guide in full accordance with the preoperative plan and ensured the restoration of patient facial symmetry.

**Lessons:**

Optimized reconstruction guide template could accurately locate the preformed reconstruction plate. This component had the ability to ensure that the location of the actual reconstruction plates were highly consistent with preoperative designed models.

## 1 Introduction

Currently, the fibula bone graft has been commonly used for mandibular reconstruction due to various advantages.^[1–3]^ The treatment not only needs to enhance patient facial appearance yet also must restore maxilla–mandibular relationships. Likewise, these methods must maintain proper reduction and fixation to restore oral and jaw function. However, mandibular reconstruction with the fibula flap can be inherently demanding and is severely limited by the technical level and clinical experience of the surgeon due to the absence of intraoperative anatomic landmarks to create a guiding and occlusal relationship. Thus, the therapeutic effect of this procedure cannot be guaranteed. Therefore, the application of new surgical techniques using the fibula bone graft to accurately reconstruct mandibular morphology, shorten operation time, and improve clinical effect has become a large dilemma for surgeons to solve.

Many studies have reported a high success rate of mandibular reconstruction with digital surgical techniques, including surgical navigation and 3-dimensional (3D) digital guide technology,^[4–6]^ which have markedly improved the precision and minimally invasive performance of modern oral and maxillofacial surgery. For instance, using a reconstructed three-dimensional skull model to pre-bend the titanium plate could assist correction of mandibular defects to a certain extent and check the effect of bond repair. Despite these recent advancements, much uncertainty remains during the segmental mandibular defect reconstruction operation even for seasoned surgeons supported by advanced technology. This phenomenon is due to the lack of an auxiliary positioning device, occlusion of bone defect segment, and landmark criteria for correct reduction. In actual surgical procedures, bone surface and reconstruction plates are hard and slippery; there is always an inevitable error between the actual intraoperative position of preformed titanium plates and that of the preoperative placement on the 3D model. Existing reconstruction guide plates are incapable of fully assisting implant fixation and titanium plate installation. Hence, if a guide template could be optimized reasonably or have the ability to correctly direct the preformed reconstruction plate position to the desired location according to preoperative design, it would reduce errors between the preoperative design and the actual operation. Surgeons would achieve accurate, personalized, and minimally invasive treatment of complex mandibular reconstruction with such a template.

In this article, newly optimized digital guide plates were designed and applied in the clinical treatment of 2 patients with mandibular ameloblastoma. We used a particular personalized “slot” to guide the preformed reconstruction plate into the target position. Image registration was used to evaluate the consistency of clinical application outcomes and our virtual surgical plan. As a result, all of the surgical guides presented showed a positive therapeutic effects both for trans-midline bilateral mandibular defect and for hemi-mandibular defects.

### 1.1 Consent

Written informed consent was obtained from the 2 patients for publication of the article and any accompanying images.

## 2 Case report

**Figure 1:**
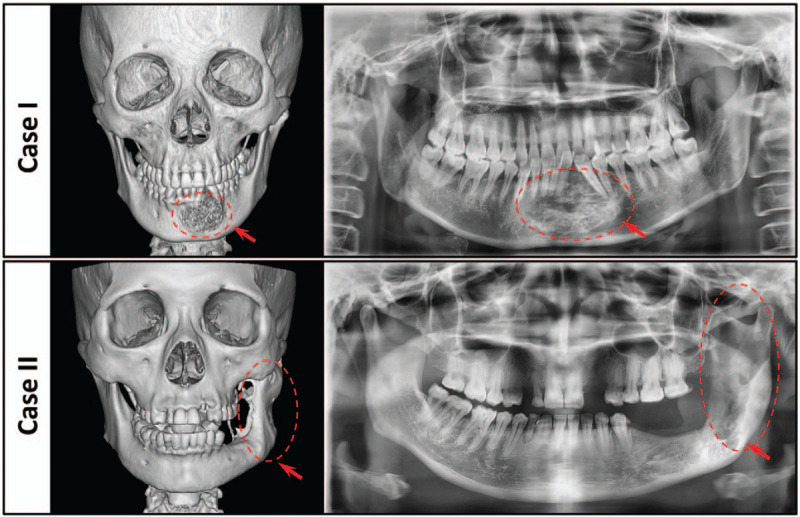
Preoperative CT images and panoramic radiographs of the patients. Case I: trans-midline bilateral mandibular defect (ameloblastoma located in the middle of the mandible). Case II: hemi-mandibular defect (ameloblastoma located in the left mandibular angle and ramus region). Red arrows and dotted lines indicate tumor areas. CT = computed tomography.

To begin our study, 2 patients with long-term expansion of the mandible were admitted to the Department of Oral and Maxillofacial surgery in the West China Stomatological Hospital, Sichuan University. Case I was a 39-year-old female patient who was diagnosed with the ameloblastoma in the middle of the mandible, Case II was a 45-year-old female patient who was diagnosed with the ameloblastoma at the left mandibular angle and ramus region (Fig. 1). In this case reports, newly optimized digital guide plates were designed and applied in the precise mandibular resection and reconstruction treatment of the 2 patients. The Institutional Ethics Committee of West China College of Stomatology, Sichuan University approved the study and the patients provided written informed consent to participate. We have read the Helsinki Declaration and have followed the guidelines in this investigation.

### 2.1 Stage I: preoperative CT-based planning

In preparation for the surgery, spiral CT (Siemens Sensation 16; Siemens, Munich, Germany) images of the head and selected fibula from the patients were obtained and stored in Digital Imaging and Communications in Medicine (DICOM) format with 0.625-mm slice thickness. This material was then imported into Mimics Research 17.0 (Mimics 17.0, Materialise NV, Leuven, Belgium).

**Figure 2:**
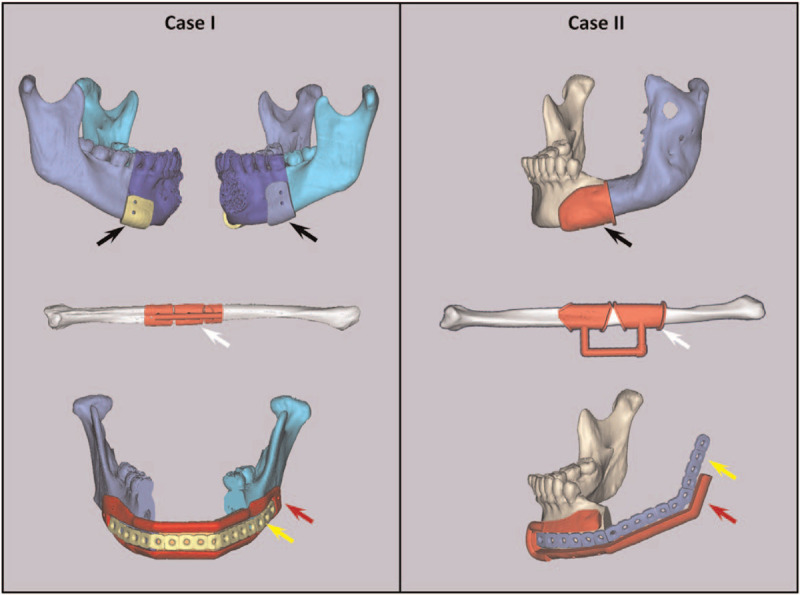
Virtual design of the surgical guide plates. Case I: trans-midline bilateral mandibular defect. Case II: hemi-mandibular defect. Black arrows: right and left mandibular osteotomy guide plates. White arrows: fibular osteotomy guide plates. Red arrows: mandibular reconstruction guide plates. Yellow arrows: preformed reconstruction plates.

Next, computerized composite skull models of the patients were generated using this program. Specifically, the entire surgery process (including tumor resection and reconstruction with the fibula flap) were simulated on 3D models (Fig. 2). Afterwards, the shape and position of the virtual reconstruction plates were preformed and designed in advance, according to the postoperative mandibular model. Subsequently, the postoperative mandibular models were imported into 3-Matic 9.0 (Materialise NV, Leuven, Belgium) software for the purpose of designing digital surgical guides.

According to the position of the osteotomy line and reconstruction plate, we marked the appropriate guide plate position on the mandible and extended it reasonably for stability. Thus, our mandibular osteotomy guide plates were designed based on the results of the virtual surgery outcome. Meanwhile, the ideal fibula osteotomy guide consistent with the length and contour of the reconstruction model was divided into 2 or 3 parts according to the osteotomy line. Two holes were designed on each guide plate consistent with an 8 mm titanium screw to help fix the guide on the mandible during the operation.

**Figure 3:**
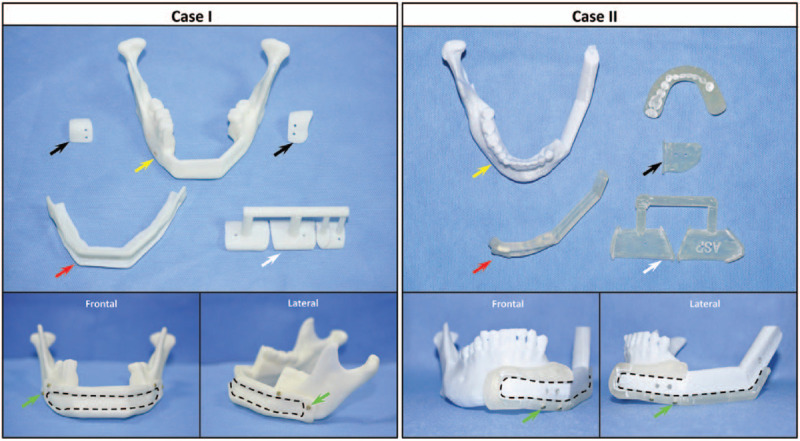
3D printed models of the reconstructed mandible and surgical guides. Case I: trans-midline bilateral mandibular defect. Case II: hemi-mandibular defect. Black arrows: right and left mandibular osteotomy guide plates. Yellow arrows: postoperative mandible models. Red arrows: mandibular reconstruction guide plates. White arrows: fibular osteotomy guide plates. The frame of the black dotted lines: “slot” for guiding the preformed reconstruction plate. Green arrows: nail holes and screws used to fix the reconstruction guide plate on the mandible. 3D = 3-dimensional.

Furthermore, special “slots” (Figs. 2 and 3) in the middle of the reconstruction guide plates were designed exactly according to the position, shape, and size of the preformed virtual reconstruction plates. The “slots” (Figs. 2 and 3) that were used for the hemi-mandibular defect were designed to be of an “unsealed” type. Accordingly, 2 titanium nail holes (Fig. 3) with a radius of 8 mm were designed on respective left and right sides of the guide to facilitate intraoperative fixation of the mandible. The inner surface of the guide was consistent with the surface of the mandible, and the lower edge of the guide was grooved to a depth of about 5 mm (consistent with the irregular contour of the mandible). The thickness of the guide was about 2.0 to 2.5 mm, slightly thicker than that of the pre-formed titanium plates.

On this basis, all surgical guides and reconstructed mandibular model files (STL format) were imported into a rapid prototyping machine (Wiiboox; JOC, Jiangsu, China) for 1:1 3D printing (Fig. 3). Next, the mandibular model and guide plates were used to preform the real titanium reconstruction plate. The guide template and actual reconstruction plate were fixed to the mandibular model with screws to ensure that they were perfectly adapted to the defect. Finally, all of the guide plates, reconstruction plates, screws, and skull model were sterilized.

### 2.2 Stage II: surgical procedure

**Figure 4:**
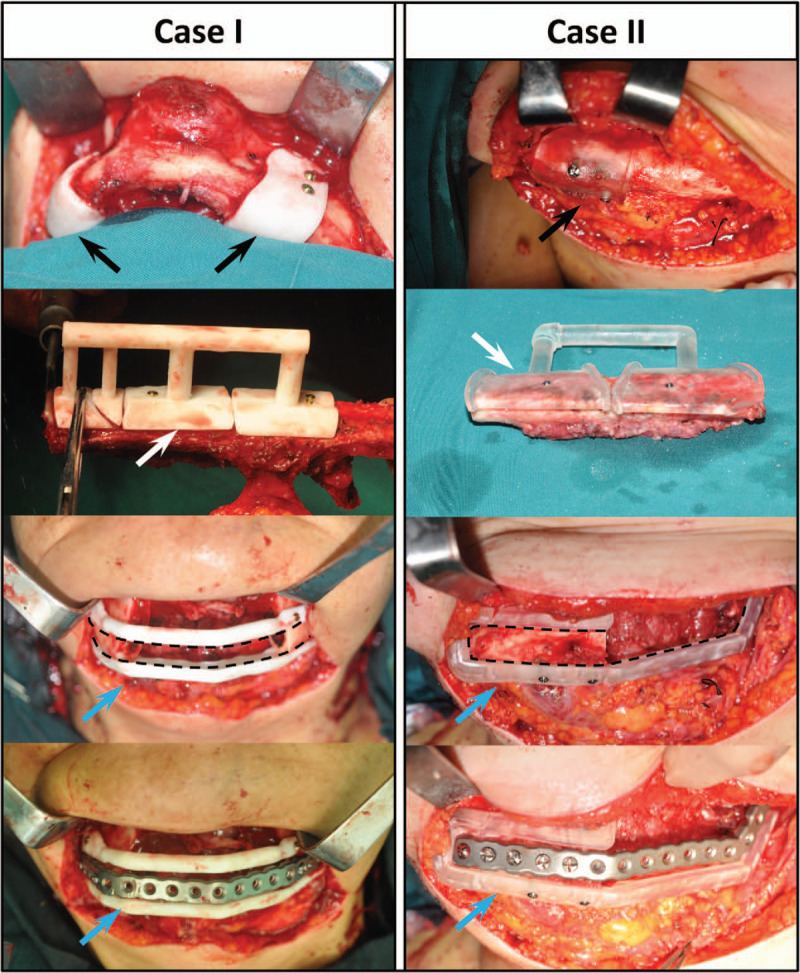
Clinical application. Case I: trans-midline bilateral mandibular defect. Case II: hemi-mandibular defect. Black arrows: right and left mandibular osteotomy guide plates. White arrows: fibular osteotomy guide plates. Blue arrows: mandibular reconstruction guide plate. The frame of the black dotted line: “slot” for guiding the preformed reconstruction plate.

For the real-time operation, the surgeons were divided into 2 groups to carry out the mandibular resection and fibula flap harvest. One group exposed the mandible through a submandibular incision and finished the mandibular resection with the help of our mandibular osteotomy guide (Fig. 4). The reconstruction guide plate was fixed onto the remaining mandible with screws according to the anatomical shape of its inner surface. This portion fixed the 2 remains of the mandible into a whole while retaining proper space for the fibular flap after the mandibular resection. The preformed reconstruction plate was then fixed to the mandible through a “slot” (Fig. 4). Meanwhile, the other surgical team cut the fibula using the fibular osteotomy guide plate. Lastly, the vascularized fibula flap was fixed to the mandible with the reconstruction plate to restore normal contour of the mandible.

### 2.3 Stage III: postoperative findings

**Figure 5:**
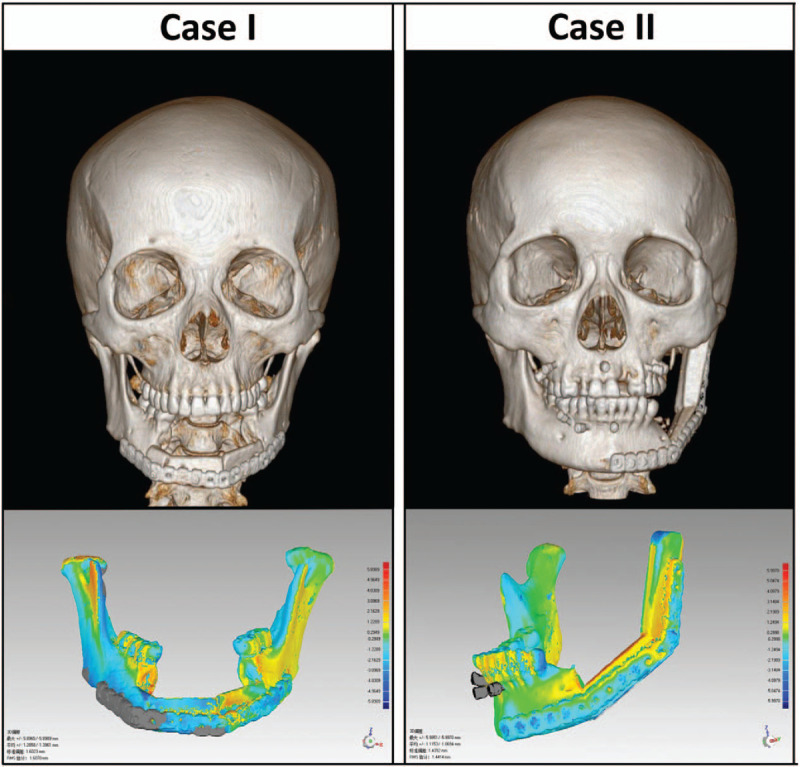
Postoperative CT and image fusion of the preoperative virtual design and postoperative 3D model. 3D = 3-dimensional; CT = computed tomography.

At their 3 months follow-up appointment, 2 patients had a pleasing facial appearance and had recovered their normal oral functions (Fig. 5). Geomagic Studio 2013 (Geomagic, NC) software image fusion technology was used to compare the actual 3D model of surgical results with preoperative planning. This information indicated that the location of the fibula segments and reconstruction plates were highly consistent with our preoperative designed models. To further this success, patients were instructed to avoid strenuous exercise, and no postoperative complications were observed.

## 3 Discussion

“Personalized, accurate, and minimally invasive treatment” is the explicit goal of precision medicine.^[7–9]^ In the past, the repair and reconstructive effects of surgery on maxillofacial tumor patients was poor, and postoperative complications such as facial asymmetry and occlusal relationship disorder often appeared. In recognition of technological advancements, many maxillofacial surgeons have attempted to treat mandibular reconstruction using assistance by digital surgical methods, especially the combination of digital guides and a preformed reconstruction plate.^[10–12]^. Digital surgical technology facilitates surgeons to comprehensively understand oral and maxillofacial defects together with making an exact preoperative plan.

Nevertheless, these prior plans sometimes failed in practice because the preformed reconstruction plates and fibula segments could not be placed accurately due to the lack of anatomical markers.^[13]^ Even worse, the reconstruction plate sometimes needed to be bent again during the operation,^[14]^ which could prolong the operation and reduce its repairing effect. Therefore, in this study, we designed a variety of reconstruction guide templates to solve the above problems. Specifically, we held the consideration to restore mandible morphology through preoperative virtual surgery design. We are attempting to transform virtual surgical planning to absolutely match the real-time operation. Since each case of mandibular defects are unique and require different surgical methods, the choice of approach and order of reduction all affect the surgical procedure. In the design processes of our digital surgical guides, we had the ability to personally optimize features. Throughout our study, a series of 3D digital surgical guides were used to lead mandibular reconstruction using fibula flaps in combination with a preformed titanium plate. To that end, commonly used mandibular resection, fibular, and optimized reconstruction guides were all designed according to surface morphology of the mandible and the fibula. In fact, the inner surface of each guide plate was perfectly matched to its chosen part. Thus, in theory, each of these plates has only one target location on the mandible and fibula, allowing surgeons to place it on the bone surface.^[11,15,16]^ In addition, our fixation nail holes could enhance the placement stability of the guides. This design greatly increased guide template position stability and reliability, as demonstrated below.

It has been proven that using a preformed reconstruction plate has many advantages relative to a manually bent plate in operation, such as fractures, tumor resection, orthodontic surgery, and implant surgery.^[6,15–17]^ However, even experienced surgeons face difficulty in placing the preformed plate in the same position as its preoperative design because of unstable bone segments and the lack of effective stabilization after the tumorectomy stage. In this project, a specially designed reconstruction guide template solved the difficulty of placing the preformed titanium plate via determinations during preoperative virtual design. We innovatively devised a “slot” in the middle part of the guide that was generated exactly according to the position, shape, and size of the preformed reconstruction plate. Its bilateral flanks ensured accurate positioning of the reconstruction guide plate on the surface of the mandible. The actual reconstruction plate was then preformed according to the “slot” shape before the operation. Moreover, after the reconstructive guide plate was fixed, the “slot” structure not only directed the placement of the reconstruction plate but also allowed the surgeon to fix it directly without removing it. In the operation, after the guide was successfully fixed on the mandible, the “slot” clearly showed the accurate position of the preformed reconstruction plate, deeming it an excellent locating device. In fact, during the operation, the surgeons only needed to insert the reconstruction plate directly into the “slot.” Hence, the combination of the guide and the preformed reconstruction plate saved time of finding correct position of the reconstruction plate. As a result, we effectively solved the difficulty of locating the preformed reconstruction plate on the mandible. Furthermore, for trans-midline bilateral mandibular defect cases, the remaining 2 parts of the mandible could receive preliminary support and retention with the guide by tightening the screws in its nails, which provide the convenience of further fixation from the reconstruction plate.

Therefore, the personalized, optimized design of “slot” played its expected role in the real-life operation. Surgeons experienced that the procedure was greatly simplified with the use of this series of guides compared with the conventional ones.^[18]^ They could easily amputate the mandible and fibula according to the designed osteotomy line with the assistance of the osteotomy guides and place the preformed reconstruction plate through the specially designed “slot” of the reconstruction guide without concern about the mobility of the remaining mandible or possible changes in the position of the temporomandibular joints. Besides, this equipment further facilitated surgeons to fix fibular bone segments. Our newly created “slot” makes it possible for the guide and reconstruction plate to work together, which could improve the effect of this treatment. The combination of our new optimized guide and the preformed reconstruction plate can provide precise treatment in mandibular reconstruction. As a result, operation time was shortened yet most importantly, the success rate and results of the surgeries were greatly improved. Two patients were satisfied with their recovered facial appearance.

Our optimized guide plates are somewhat large in volume, just as the guides used in other studies.^[18,19]^ Surgeons need to clear the appropriate bone surface to ensure placement of our guides. This design considers defects of the mandible after a large tumor resection or trauma that includes large soft-tissue incisions or wounds. No extra incisions or excessive stripping of healthy muscle attachments are needed. Thus, it is necessary to consider whether the guide plate is expedient during practical applications. In the future, the design concept of the guide plates would be readjusted for patients with small facial defects if the oral incision were proposed.

In brief, we provided one optimized guide for mandibular reconstruction, which can accurately locate the preformed reconstruction plate and force them to work together. Implementing the assistance of such a guide, we achieved accurate mandibular repair in full accordance with the preoperative plan and ensured restoration of patient facial symmetry. The digital surgical guides used in the operations reported here showed great clinical application value and further achieved surgical precision. These creations are of a simple design, time-efficient, moderately priced, and produce the ideal surgical effect.

## Author contributions

**Conceptualization:** Jie Long.

**Formal analysis & Methodology:** Lu Han, Xiaojie Zhang and Zeyou Guo.

**Writing – original draft:** Lu Han.

**Writing – review & editing:** Jie Long.
